# Book Reviews

**Published:** 1813-05

**Authors:** 


### CRITICAL ANALYSIS
### *OF RECENT PUBLICATIONS*
IN THE
### DIFFERENT BRANCHES OF PHYSIC, SURGERY, AND MEDICAL PHILOSOPHY.

---

*An Examination of the Imposture of Ann Moore, called the Fasting Woman of Tutbury; illustrated by Remarks on other Cases of real and pretended Abstinence. By* ALEXANDER HENDERSON, M.D. Physician to the Westminster General Dispensary. 8vo. pp. 52. Underwood and Black. 1813.

AS the learned author has already submitted the substance of this tract to the public, through the medium of our Journal, we have merely to announce the present improved edition. He has introduced some other similar instances in support of his former conclusions respecting the imposture of Ann Moore, although, in our opinion, he had made out a case sufficiently strong for all men, except those on whom a passion for the marvellous has produced mental darkness. The facility, indeed, with which some men, even with pretensions to sense, lend themselves to idle stories, and maintain the existence of impossibilities, is a powerful check to human pride, but of so frequent occurrence, that it creates no shame; nay, the detector of error, and the exposer of fallacy, too often encounters the scoffs and the ridicule of the many who still close their eyes against the light which would reveal the darkness of their understanding. A German enthusiast (Hans Engelbrecht) who wrote an account of his own death and recovery, and of what he had seen in the other world, declared that an angel expressly ordered him to write a full and particular narrative, and publish it. "Now," he says, "this was my motive for getting up very early this morning at four o'clock to begin; and therefore do I exhort you, all ye men in the world who get the reading of this narrative into your hands, to be sure not to suffer your reason to *perk up* and be *dictating* therein, but believe you this *simply*, just as I have written it down:" and, as a clenching argument, he concludes, "such as will not believe what I am now about to write will be damned." We will not suppose that the favorers of the eternal law of nature regarding the human constitution, being violated in the person of Ann Moore, which it must be if her account were

1 true, true, would be so merciless as to have all unbelievers of their narrative damned; but we do conceive that it would be extremely convenient to them if the injunction of brother Engelbrecht, not to allow their reason to *perk up*, were especially heeded.

Among the instances which Dr. Henderson has adduced in support of his opinion that the fasting woman of Tutbury is an impostor, is one which bears so many points of resemblance, that he has subjoined a parallel of their cases, which we shall insert for the amusement as well as edification of our readers. Anna Maria Kinker, the notorious counterpart of Ann Moore, appears to have exhibited her feats at Osnaburg.

PARALLEL *of the Cases of* ANN MOORE *and* ANNA MARIA KINKER.

| *Ann Moore.* | *Anna Maria Kinker.* |
| --- | --- |
| "Her countenance is fresh and animated."—"She is naturally of a cheerful and talkative disposition.—*Med. and Phys. Journ. and Account, &c. by J. L.* | "She had an agreeable physiognomy, and a very fair complexion: her cheeks were rather florid."—"She appeared cheerful."—*Gruner*, p. 7, 18. |
| "Possesses great strength of mind."—"This circumstance convinced me of the powers of her memory."—*Account, &c. Month. Mag.* | "She displayed great strength and quickness of memory on all occasions, and no common degree of curiosity."—P. 36. |
| "Several remarkable expressions, which have fallen from her lips, during the course of conversation with different people, tend much to show the pious state of her heart."—*Account.* | "The pious resignation with which the mother and daughter spoke of her extraordinary condition, contributed not a little to heighten the effect of the scene."—P. 7. |
| "The number of people who go to see her is astonishing; and every one giving her a trifle for the benefit of her children, she has by this time received something very handsome for them."—*Ibid.* | "There was no want of visitors, even from among the higher ranks; and they seldom took leave without making a present to the girl or her parents."—P. 8. |
| She was watched from the 13th to the 29th of September, by a number of persons selected for that purpose, who affirmed, that she had not eaten or drunk any thing during that time, except about an ounce and a half of water. | A. M. K. was watched from the 11th to the 25th of May, by six persons on oath, who declared, "that she had taken no solid food or drink during that period, nor been observed to have any evacuation."—P. 6. |
| On Mr. Thompson's proposing a second watching, she said, "that she had been upon her trial once, which | Dr. Schelver, when he intimated the design of a second watching, "was surprised to find that not only |

*Ann Moore.*

which she would not then have submitted to but to oblige the minister, and for nobody in the world would she undergo a second watching. Her attendant was pleased to style it a trial for her life."—"The young women being asked if they would consent to her being removed to another house, at first hesitated; saying that it would be very likely to kill her."—*Account.*

"She cannot endure people in the room who have taken the smallest quantity of malt or spirituous liquors: the fumes of their breath affect her in such a manner as to cause a giddiness of her head."—*Ibid.*

Of all the fingers of the left hand, except the index, she said that she had lost the use: the middle finger, indeed, she admitted could be moved by external force, though not by volition. But, when not attending to it, she was observed to use the finger in question without any difficulty.

In her lower extremities she declared that she had no feeling whatever.

"She cannot endure without a fresh current of air continually admitted into her room, for which purpose the chamber window is always open, even in the coldest weather."—*Account.*

She told Mr. Corn, "I feel no hunger or disposition for food, neither did I for many years before I declined eating."—*Month. Mag.*

When Dr. Darwin proposed to hold a mirror before her face, in order to examine her respiration, she exclaimed, "No more experiments for me! I have suffered enough already from experiments."

"She

*Anna Maria Kinker.*

only the patient, but her parents, appeared very unwilling to submit to it; although the father had repeatedly assured him that he would be glad to see a repetition of the watching, and would consent to the removal of his daughter, if she could be cured without expense to him." The father said, "It was enough that his daughter had been watched once, and that she would not be able to bear the removal."—P. 10, 20.

"She seemed to have an acute smell, for she requested us not to smoke, because she could not bear it; and she complained that the lavender-water, at which she was smelling, was too strong for her."—P. 18.

"Although she asserted that she had no feeling but in the breast and head, we remarked, that, while she was looking at us, she rolled a riband round her fingers, and also reached behind her pillow for two sheets of paper which were placed there, without being able to see them."—P. 19.

"She affirmed, that she had no feeling in her legs."—*Ibid.*

"Notwithstanding the dismal cold weather which we had at that time, she desired that the window, which had been open all the day, might be kept open also during the night."—P. 38.

"While we were at breakfast, she assured us that she loathed food."—P. 42.

"Some days before, she said, a merchant had come from Osnaburg, and told, that they wished to make experiments with her, advising her father not to consent to them."—P. 43.

"She

| *Ann Moore.* | *Anna Maria Kinker.* |
| --- | --- |
| "She sleeps well."—*Taylor.* "Her head and left side are never free from pain, so that she has no sound sleep night or day."—*Account.* | "She appeared to sleep soundly; yet she averred, that she only slumbered, and had heard all that was spoken."—P. 45. "Complained of a pain in the left hypochondrium."—P. 112. |
| She expressed her "willingness to submit to any thing that was thought necessary for the satisfaction of the public."—*Ibid.* | "She told us her relation was true, and that we were at liberty to publish it."—P. 60. |
| "She finds every good effect attained from the occasional cleansing her mouth with a moistened rag."—*Taylor.* | She confessed, "that since the second day of our watching, she had contrived to convey a certain quantity of water into her mouth, by squeezing the sponge;—that, during the first watching, she had sucked some water from a wet cloth, which was placed under the left side of her face, under pretence of heat."—P. 83-4. |
| "In the course of the first three days of the investigation, she swallowed in the whole about an ounce and a half of water; but, happening to step into the room while she was swallowing it, the extreme misery of deglutition, and the violent rising of wind resisting its passage to a degree that almost seemed to threaten suffocation, induced me to dissuade her from taking any more, while the experiment that was to vindicate her veracity continued."—*Ibid.* | Confessed, "that, during the last eight days of the first watching, her mother had secretly conveyed into her bed a small bottle of water; and that she had drunk it in the night, when the lamp was so placed that the watch could not observe her."—P. 126. "She was now made drink some mouthfuls of water, which she swallowed with affected difficulty."—P. 84. |
| "She constantly begs not to be urged to take any thing, as the attempt to swallow gives her grievous pain."—*Allen.* | She said, "that, when her father, at any time, asked her to eat, she had always begged him not to put her to that pain."—P. 150. |
| "Convulsions have come on from so slight an excitement as surprise."—*Granger.* | Confessed, "that she had two or three times counterfeited fainting, in order to deter us from attempting to move her."—P. 152. |

Our readers are now in possession of the facts which the industry and ingenuity of Dr. Henderson have enabled him to acquire upon a very curious subject. The public appears to take considerable interest in it; for, though honest John Bull is a very credulous animal, he is very angry when he discovers that a deception is practised upon him. We

 shall, shall, probably, in our next Journal, have occasion to notice a very different publication, taking a very opposite view of the subject, and embellished by the designs of one of the ablest draftsmen of the present age. We also learn, that, at the suggestion of a certain divine, Ann Moore has consented to submit to another ordeal:—we wish her well through it; for, although she has experience in her favor, the means of deceiving her guard will probably not be so practicable as on a former occasion. We would also remind those curious gentlemen upon whom her bold assertions and confident demeanor make a strong impression, of the hoax practised some years since at the Haymarket theatre, where a crowded audience actually assembled to witness a grown-up man squeeze himself into a quart pot; or of the pie-man who persuaded many sober people his delectable cry of hot! hot! on Westminster bridge, could be heard at Chelsea.

---

*Edinburgh Medical and Surgical Journal, No.* XXXII.

I. *Letter to* Dr. ANDREW DUNCAN, *jun. from* Dr. COCHRANE, *inclosing a Letter from* Dr. HARNESS, *and Communications from* Mr. PARSON, Dr. BURNETT, *and* Dr. WILSON, *on the Ardent Fever, as observed at Guadaloupe, Gibraltar, and Plymouth.*

THE object of this paper is to show the efficacy of blood-letting in the early stage of what is commonly denominated *Yellow Fever.* The continued and bilious remittent fevers of the West Indies, Mr. Parson considers but as modifications of the same disease; the predisponent causes to which are, in his opinion, "*marsh miasmata*, the consequence of high atmospherical temperature, with moisture from putrid vegetable exhalations, with which the system of a newly-arrived European being duly impregnated, a very slight exciting cause is sufficient to set this violent stimulus in motion, the first effect of which is demonstrated by an inordinate action of the heart and arteries, but a non-correspondent one in the venous and absorbent systems, which are from the commencement of the disease in a state of torpor.

"The morbid force is of a most violent and concentrated character, exhibited in the peculiar determination of blood to the brain, in most instances, but in all to the abdominal viscera in particular, where, upon dissection, the most formidable traces of the disease are always present. Its natural course, in the primary stage, consists in strong and rapid movement, ultimately tending to the destruction of certain organs, by producing congestion and inflammation.

"The curative indication consists in unloading the vessels as as quickly as possible, by copious and repeated blood-letting, until the symptoms caused by the unequal action of, and between the arterial and venous systems abate. These powerful means are to be aided by the free use of cathartics, so as to keep up a constant action of the bowels, by tepid diluent drinks, and warm baths, and, what I consider would be still better, vapour-baths, to cause a determination to the skin."

To the employment of cold affusion in the first attack of this fever, our author objects, but considers it to be serviceable after due depletion by the lancet. Of mercury, which has been so highly extolled, he says, "a very full and just trial of this mineral, and its almost total failure with me, obliges me to declare, that my faith in its efficacy in this fever, is completely shaken; and I very much doubt if it ever has effected any cure, after congestions have once taken place. Indeed, so remarkably torpid are the absorbents, during the continuance of this disease, that I equally doubt if any part of the mercury is absorbed, until the disease has been greatly moderated by more effectual means."

In some instances, the early phenomena of this disease seem to forbid depletion by the lancet; and which phenomena depend, in the opinion of Mr. Parson, on an increased impregnation of the subject with the pestilential marsh miasmata.

"Instead of finding on the first attack (in these cases) great heat of the skin, and strong arterial action, with the usual determination to the head, the surface of the body is constricted, the pulse small, and in some instances hardly discernible, though upon pressure tense and corded, coolness of the extremities, with the action of the heart in a manner suspended. These symptoms, with their concomitants, increased anxiety, &c. indicate, I think, a higher degree of disease, a larger determination of blood to the internal organs, with an increased disposition to torpor in the veins; and the circumstance of the pulse always rising after such bleeding, with the efficacy of purging, &c. are sufficient proofs of this opinion being well founded."

Under these appearances, which would certainly lead those, who had not a specific experience for their guide, to a contrary practice until an apparent re-action had taken place, it is satisfactory to have the authority of one accustomed to treat this formidable malady. We are fully of opinion, that, in a variety of forms of the febrile state, and of increased arterial action under many dissimilar circumstances, a resort has injudiciously been had to stimulants and tonics with a view to obviate subsequent debility: and that thus many morbid indications have been pushed to a fatal or to a hazardous zardous zardous point, which by an opposite treatment would not have been dangerous. Alcohol, wine, opium, and bark, have much to answer for.

Of this practical paper, the utility of which we can observe through the mistiness of an involved style, we cannot take leave without stating, that Mr. Parson has found the *Liquor Arsenicalis* greatly serviceable in remittents and intermittents. "Its use with me," he says, "in those diseases *has been unboundedly successful, particularly where the bark has failed.*"

The remaining part of this paper, which is divided into Nos. 2 and 3 of this Journal, relates particularly to the epidemic fever which occurred at Gibraltar, at Alicant, and Carthagena, in 1804, and the subsequent years; and to the treatment of the contagious fever among the seamen that were sent ashore to the Royal Hospital at Plymouth.

IV. *On Nervous Affections, and on the Treatment of Chorea Sancti Viti. By* David Uwins, M.D.

Dr. Uwins premises, with great propriety, some remarks on the folly of making all practice submit to theory; and upon the hazard now incurred by employing the evacuating plan to an absurd extent, in opposition to the tonic and stimulant hypothesis, which lately lorded over our understanding with such absolute tyranny.

Several cases of Chorea are related as treated by different and opposite methods. In the first, where wine and tonics had been employed, with violent aggravation of the disease, purging was successful. In the second, a young female of strumous idiosyncrasy, cathartics were given for three weeks, with an increase of involuntary motions. This was cured by the *argenti nitras*, given, at first, in a sixth part of a grain, but gradually increased to nearly five grains in the course of the day. The third case was in a female in her 13th year, who was afflicted with chorea approaching to epilepsy. The countenance indicated much irritability; the pupil was exceedingly dilated; the head was sometimes in great pain; and the whole body was agitated to such a degree, that, in whatever posture she might be, it was always necessary for at least one person to hold and restrain her. Calomel and purges were employed. The discharges from the bowels were considerable, of that kind which are seen as attendant on worms, and occasionally large quantities of scybalæ. The continuance of this plan for some weeks did not moderate the disease, which was afterward subdued by the administration, two or three times a-day, of the following powder:

R. Pulv.

℞. Pulv. Digitalis, gr. iij.

Ammoniæ Cupri, gr. iv.

Pulv. Myrrhæ, ʒi. fiat Pulvis.

In the fourth case the *Tinctura Digitalis* was given to the quantity of thirty drops every six hours, beginning with ten, with evident advantage as to moderating the involuntary motions. Great debility succeeded to the use of this remedy, which, after the subsidence of the chorea, was removed by bark and steel. The fifth case, which came suddenly on a fine girl of eighteen, terminated fatally in a month, with apparently serous effusion in the brain.

The result of these cases shows, in the ratio of three to one, that the treatment opposite to that of constant purging, is most to be depended on.

V. *On the Cure of Tetanus by Opium and the Warm Bath.* *By* Thomas Christie, M.D.

The intention of Dr. Christie is to recal practitioners to the old, and, in his opinion, efficient remedies, opium and the warm bath, in preference to trusting to purgatives and cold affusion, which he considers as more doubtful, though recommended on high authority.

The case of M'Gowan, related by Dr. Christie in this paper, is given with minute accuracy by Mr. Leath, the gentleman under whose immediate care it was placed, in one of the preceding numbers of our Journal, and to that we refer our readers.

VI. Dr. Adams's *Answer to* Mr. Edmonstone, *respecting the Venereal Disease at Otaheite.*

As this paper is written only with the view to defend some opinions of its author against the remarks of Mr. Edmonstone, we cannot extract any thing from it that will interest our readers.

VII. *A Letter on the Cure of Curved Spine. By* B. T. Burroughs, Surgeon.

In the two varieties of curved spine, the one arising from debility, and the other from disorganisation of the vertebræ, Mr. Burroughs reprobates the constant confinement of the patient to a horizontal posture. The object of enforcing the horizontal posture is to take off the superincumbent weight from the morbid part: but this the writer thinks is more than counterbalanced by the weakness which ensues on the long confinement to bed, with the loss of air and exercise. Where the body of the vertebræ is diseased, and the curvature results from this, the cure is asserted to take place by anchylosis; but the horizontal position, and the wearing of instruments, both tending to leave an open space which the dissolved dissolved vertebræ had occupied, prevents or retards this anchylosis, in the opinion of Mr. Burroughs. A diagram is given to show this; but so unfortunately designed, or so carelessly cut, as to exhibit the contrary to what the author means.

If we cannot agree fully with the reasoning of this writer, we accord with him in earnestly desiring the opinion of the gentleman to whom he appeals: medical science is always benefited by investigations of Mr. ASTLEY COOPER.

VIII. *Observations on the Yellow Fever of New York in* 1803. *By* ALEXANDER RAMSAY, M.D.

On this subject of great interest Dr. Ramsay offers some facts that may be useful in the completion of its natural history.

On the weakened or torpid state of the lymphatic system, which we had occasion to notice in the first article of this analysis, as so often occurring in the bilious remittent *inter tropica*, and on the use of mercury in this disease, Dr. Ramsay expresses himself as follows:

"The diseased state of the lymphatics (which seems always connected with debility), which I discovered and described when a pupil of the celebrated Mr. Cruickshanks of London, was detected in several of those subjects who died of yellow-fever. This consists in the lymphatics losing their transparency and elasticity, and assuming an opake and inelastic state. In proportion as this opacity increases, so does their dilatation and thickening of the coats; the utility of the valves ceases; congestion takes place. When such vessels suffer compression, or when they cease at any time to be filled with their contents, the highly active state of the vasa propria occasions accretion of the inner coat, and obliteration of the cavity, by which the regular progress of the lymph is prevented. This affection of the lymphatics seems most frequent in the lungs, and not unfrequently in the iliac plexus. It seems induced by any and all debilitating causes, especially indiscreet mercurial applications; and I attribute those I met with in America to this cause, as mercury is the incessant resort of practitioners in the interior of that country. I believe much harm is occasioned to the general constitution by the indiscriminate use of this medicine, in the northern states in particular; and I was pleased (and indeed honored) to find, that the opinions of the highly-respectable Dr. Saunders, of London, corresponded with the notions I had given (in the colleges of New York, &c.) in his late judicious remarks on this very subject. This affection of the lymphatic system, (since I have divulged it to practitioners,) seems very prevalent in all parts of the human body. One of the first practitioners in midwifery in London, informed me, that for many years he had discovered the appearances mentioned in diseased mammæ, but till then was at a loss to what cause to ascribe it. In indolent tumors, this seems to have been traced as the foundation of the evil."

 Several

Several proofs are given of the endemic quality of the yellow fever, which afford negative evidence of its non-contagious nature.

The history of what are called *walking cases*, is a remarkable feature in this fever.

"The person appears yellow, is listless, often seemingly in a state of fatuity; sometimes you cannot attract his attention by addressing yourself to him; he walks about, without design, from place to place, and, in a moment, drops lifeless to the ground, without any previous symptom to warn attendants."

IX. *Case of Pseudo-Syphilis, with Remarks. By* WILLIAM STEVENSON CLARK, Surgeon.

That diseases resembling syphilis have appeared at all periods, and that medical science is indebted to Mr. Abernethy and Mr. Pearson, for elucidations of those obscure, intricate, and often doubtful, maladies, will not now be controverted. As the comprehending a series of morbid actions under any *specific title* is facilitated by detail, we shall make no apology for transcribing Mr. Clark's interesting case verbatim.

"I was consulted some time ago by a person on account of an excoriation on the side of the prepuce. The history of the case was this: His health had been for some time rather impaired: in the early part of January he had a connection; from this, however, no visible morbid symptom arose, till some weeks after, when a slight irritation was felt upon the prepuce; this was soon followed by a sore, extending from the middle of the left side of the prepuce, to the frænum, and succeeded by an enlargement of the glands in each groin. Upon viewing accurately the sore, the surface of which exhibited little of the characteristic marks of a true venereal chancre, and particularly attending to the *progress* of the case, and the *length* of *time* that had elapsed between the connection and the appearance of the sore, I pronounced it not to be venereal, and directed the most simple applications to be made use of, as a saturnine lotion, or a poultice of milk and bread. This plan was tried for some time, when it appearing to make no alteration, at least for the better, I recommended a mild solution of calomel in lime-water, which in a short time completely healed it. Nothing now whatever appeared till early in May, when I was again consulted by the same person, on account of an uneasiness in his throat. Upon examining the tonsils, I distinctly perceived a slight ulceration on each, accompanied with a degree of erysipelatous redness. The ulcerations kept gradually, but superficially, spreading, for about a week, when an eruption of purple spots took place upon the skin, at the same time the inguinal glands continuing greatly enlarged. Confident, however, partly from the appearance of the primary sore, but more particularly from the *history* of its *progress*, that it was not venereal, I recommended the use of mild detergent gargles, and a moderate quantity of red port daily. From this time an uniform increase of the symptoms took place; the eruptions became more vivid, and the ulcerations of the tonsils, tonsils, although extensive and sloughy, yet did not appear to penetrate deep, or to assume the thickened ragged edge. The case now became of considerable importance, and had arrived at a crisis which rendered it necessary for some additional steps to be pursued. I accordingly recommended the warm bath, which was to be repeated as often as the patient's strength would permit, and advised a plentiful use of the Peruvian bark, together with the application of the oxymel æruginis to the tonsils. From this period to the beginning of July, a slow and almost uninterrupted progress was made. The ulcerations had extended from the tonsils up the inferior edge of the velamen palatinum, at which time the tonsils assumed a disposition to heal, but again quickly ulcerated. No benefit having at all resulted from the means hitherto made use of, and his health beginning to suffer, I determined that he should repair into the country. The symptoms, which had hitherto been characterised by a very slow progress, now assumed a much more rapid increase; the ulcerations spread on one side of the velamen palatinum, crossed the uvula, and completely affected the other, and the copper-colored eruptions became larger and more universally diffused. The conviction, however, of the peculiar character of the primary symptoms still supported me in the belief, that, although a most obstinate poison was secreted and thrown into the system, yet, that it was essentially different in its true specific properties from the venereal poison. In this opinion I had shortly the gratification of being confirmed, for a fortnight had not elapsed before a rapid and total disappearance of the eruptions took place;—this is a character which most decidedly marks the spurious syphilis from the genuine, as will be shortly observed at the conclusion of the case. In a few days the eruptions again appeared, and continued for the space of three weeks, at which time the progress of the poison appeared to have arrived at its acme. I now thought it right to try the effects of the sarsaparilla, and, for this purpose, directed half a pint of the compound decoction of sarsaparilla to be taken three times a-day, combining with each dose from five to eight drops of the muriate of barytes. From this period the ulcerations in the throat began to subside, their sloughy surface to look more healthy and to granulate, and the eruptions gradually to disappear, except some slight fluctuations which have since taken place, sometimes all the symptoms being nearly exhausted, and then appearing again to resume a fresh morbid action, till the present time, when they have completely disappeared. Thus has a most interesting case, which lasted, with very ambiguous symptoms, from the beginning of May to the latter end of September, finally terminated *without one single particle of mercury*, and in a constitution where probably a complete course of that mineral would have proved fatal."

X. *Further Observations on Painful Subcutaneous Tumor.* *By* William Wood, Surgeon.

To the valuable histories related in a former paper on this subject, a full analysis of which will be found in one of our preceding numbers, Mr. Wood has here added two more cases. The practice of extirpation has in those also been successful.

The

The object of Mr. Wood is, more particularly, to give in this paper a detailed review of what had been previously known on this subject. The first mention of this disease is found in Cheselden's Anatomy, 10th edition, who had thrice met with it. Camper, in his *Demonstrationum Anatomico-Pathologicarum, liber primus,* describes a species of tumor not larger than a pea, as giving rise to severe pain, and requiring extirpation for its cure. In the third volume of the Memoirs of the Medical Society of London, Dr. Bisset gives a minute detail of a case of this disease; and in the sixth volume of Dr. Simmons's Medical Tracts and Observations, Mr. John Pearson relates a case of this complaint, with several ingenious remarks.

XI. *A Case of Purpura Hæmorrhagia. By* THOMAS JEFFREYS, M.D. of Liverpool.

A captain of a Swedish merchantman, aged 40, of a robust habit, and inured to hardship, applied to Dr. Jeffreys on account of an "effusion of spots of a logwood color, profusely sprinkled upon his arms, thighs, and some on his body, differing in size from that of a pin-head to a pea; the same also appeared on his lips, and within his mouth, one of which was as large as a horse-bean, elevated and spongy, but free from pain; his gums occasionally bled; no fever; his appetite and spirits good. He had been the subject of these symptoms, more or less, for several weeks, and had used different local remedies by the advice of a physician, who, with himself, supposed them connected with a syphilitic taint; he has repeatedly had epistaxis to a great extent, and it has annoyed him in travelling from Hull to this town." A detail of the treatment is given, which was bleeding and purging, till toward the conclusion, when Cinchona and Tinct. Ferri Muriat. were substituted. Dr. Jeffreys doubts, however, if the disease were not a case of Scorbutus petechialis rather than Purpura.

XII. *Cases of a peculiar Disease of the Testis, with Observations. By* THOMAS LITTLE, M.D.

These cases, two in number, consisted of an ulcerated state of the testis, with protrusion of fungus from the substance of the gland. Escharotics of various kinds were applied, and in the first case ligature to the protruding tumor facilitated the cure. The second case, evidently connected with a syphilitic taint, yielded to a course of mercury, with the application of escharotics to the fungus. Dr. Little was essentially directed in his practice by the observations on a similar disease by Mr. Lawrence, in the 15th number of the Edin. Journal.

*A Practical Treatise on Cataract. By* JOHN STEVENSON, Surgeon, &c. 8vo. Lond. 1813. pp. 123, and a Plate.

THIS treatise is divided into seven sections, describing the nature and seat of the true cataract—its symptoms—the liability of both sexes and all ages to that disease—the proximate cause of cataract—the exciting causes—the several modes of treatment—and on the removal of the different species by the process of absorption.

Consonant to the methods we have adopted, as most likely to make our readers acquainted with the subject matter of new publications, and to estimate for themselves the value of that matter, we shall give a short analysis of each section of this pamphlet in the order followed by the author.

The first section, *on the Nature and Seat of true Cataract*, contains a short history of the progress of knowlege respecting this disease from Galen to Richter, a translation of whose treatise on the Extraction of the Cataract, from German into English, was published in 1791.

The *Symptoms of Cataract*, the subject of the second section, are divided into external or visible, and into internal or occult. The latter, or internal, as occurring prior to visible change in the lens, are first described.

"The earliest internal symptoms of the incipient disease, and which are experienced by the patient antecedently to any opacity being perceptible in the pupil, are a slight sense of imperfection of sight, together with a settled mist before the eyes, which incessantly obscures all objects, and confuses those that are minute. The constancy and fixedness of this mist serve to distinguish the complaint from many occasional and transient *obumbrations* of vision, arising from hysteria, and sympathy of the eye with a disordered stomach; as well as from those other ocular *hallucinations*, the result of a derangement in the functions of the optic nerve, or its medullary expansion. The affected eye becomes generally, at an early period, myopic, seeing near objects only with *perspicuity*, the more distant appearing as if enveloped in a cloud or thick fog.

"Of the external or visible symptoms of cataract, a haziness or muddiness is first discoverable in the centre of the pupil, situated some way behind the iris, around which there is a black ring, encircling the more or less opake nucleus of the lens. From the centre the opacity gradually extends to the edge of the crystalline, the imperfection in vision going on in nearly the same proportion. As the opacity increases, the fore part of the lens becomes more conspicuous, which led the ancients to believe that the cataract actually approaches toward the pupil. This is, however, a delusion, the cataract remaining stationary. The opacity assumes a variety of tints, from the palest azure to a milk-white color, but which are by no means to be depended upon as true criterea of the state and consistence of the diseased lens. In some few instances, so inconsiderable is the shade presented presented by the cataract, and so nearly does it resemble the natural aspect of the pupil, that the greatest experience is indispensable for the accurate discrimination of that form of the disease."

To this abridged account of the characteristic marks of the approach to and actual existence of cataract, we have only to add that a detailed case of a disease mistaken for cataract, Mr. Hey's account of a disease liable to be confounded with cataract, and some observations on what Baron de Wenzel and others have denominated black cataract, occupy the remainder of the section.

The third section, *on the Liability of both Sexes and all Ages to Cataract*, affords very little more than the observation, according to the author's experience, that the disease happens more frequently to the male than to the female sex. Though it is allowed that cataract appears most frequently in persons passed the meridian, yet children are not exempt from it, but are even born with the disease.

*The proximate Cause of Cataract*, is the subject of the fourth section. Inflammation has been suspected of producing this state of the crystalline; but as cataract often arises without the presence of any symptom indicative of the existence of inflammatory action, that opinion is left in great obscurity. The hypotheses of Maître Jan, and St. Ives, are shortly examined, and the section concludes with a conjecture of the author's, "whether a consideration of the chemical composition of the crystalline, might tend to assist in the elucidation of this point. As the lens, according to Fourcroy, consists of albumen with a small portion only of gelatine, may it not, when deprived of *vitality*, and subjected to the temperature of the human body, undergo an alteration analogous to coagulation?" Does our author mean that the crystalline, when in the cataractous state, is absolutely dead?

*The exciting Causes of Cataract*, treated of in the fifth section, are not very lucidly displayed. Blows, wounds, exposure to strong heat, and vivid light, are enumerated as being properly exciting causes; but the section, short as it is, is principally occupied by observations on constitutional diseases, struma, scorbutus, syphilis, and gout, as giving rise to this disease, and on a peculiar idiosyncracy or hereditary disposition to opacity in the crystalline.

The sixth section gives an historical detail of *the several Modes of treating Cataract*. These are considered under the heads of dioptrical, physical, (medical we suppose the author means) and chirurgical. The dioptric means are confined to the employment of concave glasses. The physical means are are remedies internally exhibited, or externally applied. The internal remedies are those which are believed to induce an increased action of the absorbents; the external are the powerful stimulants electricity, galvanism, æther, infusion of capsicum, solutions of hydrarg. muriat., &c. But these failing, as they generally will, chirurgical aid is resorted to. The radical cure of this disease is effected by removing the opake crystalline from the axis of vision. Couching or depression, the primitive mode of removing the opake body, is noticed from its inventor Celsus, to the accident that gave rise to extraction. This accident did not, however, happen "about the middle of the last century," that is 1750, as our author says, because "M. Mery recommended, in the year 1707, the practice of extraction in all cases of the disease." We mention this merely as a *lapsus:* we may be allowed to say that a book is not worse for being free from such slips.

A third method, of modern invention, the detail of which occupies above half of this treatise, and constitutes the seventh and last section, is *the Removal of the different Species of Cataract by the Process of Absorption.* To the late Mr. Saunders society is indebted for the developement of the principle on which this operation is founded, but subsequent practitioners have improved the methods of operating, and by practice have arrived at a manual dexterity, which, perhaps, the discoverer never attained. Among these practitioners, Mr. Stevenson is emulous to stand forward as the meliorator of the practice resulting from a knowledge of the solvent properties of the aqueous humor of the eye; and, in this section, gives a minute but we think a rather desultory account, interspersed with cases, of his particular methods. The nature of our publication precludes a minute detail of these methods, but we presume they will be read in the author's work by all who are peculiarly interested on the subject. Under these circumstances, we shall be content with giving a valuable fact, and with copying the author's concluding paragraph.

The belladonna was known to our excellent naturalist Ray, to be capable of producing some extraordinary effects on the eye: among these the dilatation of the pupil was the most remarkable. This valuable fact remained without any practical appropriation until 1798, when Reimarus suggested its utility in operations for cataract; and Grasmayer, of Hamburgh, we believe, has the honor of having first employed it with that intention. The advantages to be derived from this property in the atropa belladonna, were soon after after confirmed by Mr. Paget, of the Leicester Infirmary. Since this period (1800) its frequent employment has ascertained the extent of its powers, and given dexterity in its application. It might be presumed that other vegetable narcotics did possess similar properties, and Mr. Stevenson has fully ascertained the fact. He says (p. 91) "*that a strong infusion of stramonium (the Datura S. we suppose) instilled into the eye, is equally effectual in causing a dilatation of the pupil, with the advantage too of exciting infinitely less irritation and uneasiness, than the belladonna used in the same way.*" In another place, p. 121, it is also asserted that Hyoscyamus produces a similar effect. The concluding paragraph contains an acknowledgment of the source from whence the author derived his principle, a summary of his improvements, and a declaration of the bold buoyancy of his expectations.

"The reader will perceive, (says Mr. Stevenson) by the extracts given from Mr. Saunders's letters, and my subsequent remarks, that the improvements I have ventured to offer, so far from being inconsistent with, have naturally resulted from, a practical application of his suggestions. They consist principally of a more convenient mode of producing a dilatation of the pupil; a new-invented speculum for supporting the upper eye-lid, and steadying the globe; such an alteration in the needle as gives the operator a more determined power over the common cataract; and a knife, better adapted to the particular forms of the disease. It will, I flatter myself, be admitted that there is no kind of cataract, which, by one or other of the foregoing processes, may not be reduced to a state fitted for spontaneous absorption; lenticular by means of the needle, and even dense and elastic capsular by the knife."

In going through this pamphlet, we have been struck with the coincidence of the language in many instances, and the analogy of the facts in others, which it holds with a treatise published in 1812. We had thought it our duty to offer some explanation of this; but, on the trial, we felt our incompetence to enter on that explanation in a way that would at once be agreeable to the author and satisfactory to our readers.

---

*A Synopsis of the various Kinds of difficult Parturition.* By Samuel Merriman, M.D. Lecturer on Midwifery, Physician-Accoucheur to the Middlesex Hospital, and to the Westminster General Dispensary. 12mo.; sewed. 1813.

In this synopsis Dr. Merriman divides labors into two classes:

1. Natural labor—Eutocia—*Partus Facilis.*

2. Difficult labor—Dystocia—*Partus Difficilis.*

With

With the first class we have little to do. The second comprehends seventeen orders: of these we extract the first as a specimen.

I. *Dystocia Diutina.*—Labor, the head presenting naturally, terminated without danger to the mother, principally by the pains alone, but occupying a space of time exceeding twenty-four hours.

*Dystocia diutina* is usually attributable to one or more of the following causes:

1. Original or acquired weakness of constitution in the mother, producing inert, or irregular, or partial, action of the uterus.

*Dystocia à Debilitate.*—Sauvages' Nosol. Cl. 7, O. 26, § 1.

2. A rigid and undilateable state of the os uteri, and other parts concerned in the process of parturition.

*Dystocia ab Angustiâ.*—Sauvages, § 4.

This is a very common cause of delay in first labors, particularly if the patient be above the age of twenty-seven years.

3. Small size of the pelvis, or a very slight degree of distortion.

*Dystocia ab Angustiâ.*—Sauvages.

4. Extreme distension of the uterus, from an excessive quantity of the liquor amnii.

5. Too early an evacuation of the liquor amnii, whether artificially or spontaneously produced.

6. Sudden and violent emotions of the mind.

*Dystocia à Pathemate.*—Sauvages, § 3.

7. The head of the fœtus being unusually large, or too much ossified.

*Dystocia à Mole Fœtus.*—Sauvages, § 5.

8. Monstrous formation of the fœtus.

9. Fœtus being dead:

*Dystocia à Fœtu Mortuo.*—Sauvages, § 6.

Not necessarily a cause of dystocia.

10. The funis umbilicalis being too short:

Not usually a cause.

11. Obliquity of the womb.

See *Dystocia Ectopica.*

12. Mismanagement of the early period of labor.
   a. by injudicious and unavailing attempts to give assistance.
   b. by the injurious practice of giving cordials and strong drinks, under a false idea of supporting the patient's strength.
   c. by allowing the bladder to become over distended, or by not timely opening the bowels.
   d. by the improper exhibition of opiates.

The remaining orders are arranged with equal clearness and discrimination. Dr. Merriman proposes to publish a series of cases in midwifery, in the order of this arrangement of labors: from his scientific knowledge of this department of the profession, and his extensive opportunities of acquiring facts, we anticipate an important and valuable publication. In the mean time we recommend his practical axioms axioms on the management of labors to the attention of our readers.

" 1. All attempts to hasten the progress of natural labor, by artificially dilating the parts, by prematurely rupturing the membranes, or by urging the woman to *bear down* forcibly before dilatation has taken place, tend to retard the delivery, and frequently render that labor tedious and difficult, if not dangerous, which would have terminated speedily and safely if left to nature.

" 2. If care be taken to guard against fever, and to keep the woman's spirits calm and undisturbed, labor may continue for many hours, or even for days, without hazard.

" 3. It is *very* rarely proper to rupture the membranes artificially, especially with first children. There is seldom danger either to mother or child while the membranes remain entire.

" 4. No instrument ought, on any account, to be employed without the knowledge of the patient or her friends.

" 5. The greatest deliberation should be exercised before we have recourse to an operation, always incompatible with the life of the child.* It will, therefore, be generally expedient to have a consultation with some experienced practitioner, before the employment of the perforator is resolved on.

" 6. When it becomes necessary to turn the fœtus in utero, it is of importance to bring down both the feet into the vagina; *to effect the turning slowly;* to draw down the feet along the belly, and not over the back, of the fœtus; and so to direct the body of the child, that its face shall fall into the hollow of the sacrum.

" 7. In cases of the presentation of the funis umbilicalis, it should be fully ascertained that the child is living before an attempt is made to turn and deliver by the feet. In presentations of the funis, turning is had recourse to *solely* for the purpose of saving the *child's* life; but if the child be already dead, or, supposing it to be alive, if the chance of preserving it be very trifling, we ought not, by performing this operation, to run the risque of injuring the mother.

" 8. When uterine hæmorrhage occurs, in consequence of the placenta being attached over the *os uteri,* it will become necessary to introduce the hand, turn the child, and deliver by the feet; and this operation must be performed before the woman's strength is too much exhausted.

" 9. If the hæmorrhage arise from any other cause, this operation will likewise sometimes become necessary; but frequently the hæmorrhage, in this case, will be suppressed by merely rupturing the membranes. All cases of uterine hæmorrhage require to be very closely watched.

" 10. In all cases of midwifery, precipitation is to be avoided. Whatever is done in a hurry is rarely well done. Whether, therefore, we are only introducing the finger to ascertain the presentation of the child, or to judge of the progress of the labor, or are executing the more difficult operations of delivering with instruments, of turning the child in utero, or of extracting the placenta, we should 'give our heads time to direct our hands.' "

---

* *Nulla unquam de Morte hominis Cunctatio longa est.*—Juvenal, Sat. vi.

